# A Novel Role of the Sp/KLF Transcription Factor KLF11 in Arresting Progression of Endometriosis

**DOI:** 10.1371/journal.pone.0060165

**Published:** 2013-03-28

**Authors:** Gaurang S. Daftary, Ye Zheng, Zaid M. Tabbaa, John K. Schoolmeester, Ravi P. Gada, Adrienne L. Grzenda, Angela J. Mathison, Gary L. Keeney, Gwen A. Lomberk, Raul Urrutia

**Affiliations:** 1 Laboratory of Translational Epigenetics in Reproduction, Department of Obstetrics and Gynecology, Mayo Clinic, Rochester, Minnesota, United States of America; 2 Department of Laboratory Medicine and Pathology, Mayo Clinic, Rochester, Minnesota, United States of America; 3 Epigenetics and Chromatin Dynamics Laboratory, Department of Medicine, Epigenomic Translational Program, Center for Individualized Medicine, Mayo Clinic, Rochester, Minnesota, United States of America; Baylor College of Medicine, United States of America

## Abstract

Endometriosis affects approximately 10% of young, reproductive-aged women. Disease associated pelvic pain; infertility and sexual dysfunction have a significant adverse clinical, social and financial impact. As precise disease etiology has remained elusive, current therapeutic strategies are empiric, unfocused and often unsatisfactory. Lack of a suitable genetic model has impaired further translational research in the field. In this study, we evaluated the role of the Sp/KLF transcription factor *KLF11/Klf11* in the pathogenesis of endometriosis. *KLF11*, a human disease-associated gene is etiologically implicated in diabetes, uterine fibroids and cancer. We found that KLF11 expression was diminished in human endometriosis implants and further investigated its pathogenic role in *Klf11-/-* knockout mice with surgically induced endometriotic lesions. Lesions in *Klf11-/-* animals were large and associated with prolific fibrotic adhesions resembling advanced human disease in contrast to wildtype controls. To determine phenotype-specificity, endometriosis was also generated in *Klf9-/-* animals. Unlike in *Klf11-/-* mice, lesions in *Klf9-/-* animals were neither large, nor associated with a significant fibrotic response. KLF11 also bound to specific elements located in the promoter regions of key fibrosis-related genes from the Collagen, MMP and TGF-β families in endometrial stromal cells. KLF11 binding resulted in transcriptional repression of these genes. In summary, we identify a novel pathogenic role for KLF11 in preventing *de novo* disease-associated fibrosis in endometriosis. Our model validates *in vivo* the phenotypic consequences of dysregulated Klf11 signaling. Additionally, it provides a robust means not only for further detailed mechanistic investigation but also the ability to test any emergent translational ramifications thereof, so as to expand the scope and capability for treatment of endometriosis.

## Introduction

Endometriosis is a common, debilitating disease that affects approximately 10% of predominantly young, reproductive-aged women [1], [2]. The disease causes long-term morbidity from chronic pain, infertility, pelvic-organ dysfunction and combinations thereof, thereby adversely affecting quality of life [3]–[5]. Endometriosis also has a significant economic impact, in excess of 20 million dollars annually, as a result of both direct treatment costs and indirect expenses incurred from work-absenteeism [6]. As endometriosis is usually responsive to sex steroids, conventional therapies either enhance or abrogate hormonal stimulation. However, such an approach can be unfocused, often resulting in dissatisfaction with treatment and undesirable side effects. Likewise, the scope of surgical treatment is also limited by a high recurrence rate, which may eventually lead to extreme measures such as removal of the uterus and ovaries. Endometriosis is the third most common indication for a hysterectomy, often at a young age [7]. There is therefore a need for focused mechanistic research that can be translated into expanded therapeutic capability for this common and widely prevalent disease.

One of the major limitations in endometriosis research is the paucity of robust animal disease-models. Ideally, a disease-model should phenocopy human disease, while also offering the capability of scientific investigation into the effects of both intrinsic (e.g. genes) as well as extrinsic (e.g. environment) factors on disease progression. The commonest laboratory animal models with such capability are mice; however they do not spontaneously develop endometriosis. This is likely from a lack of menstrual cycles and therefore also of retrograde menstruation, which is the most widely accepted etiological theory of endometriosis. Endometriotic lesions have however been successfully implanted and studied by several groups using two basic types of mouse models. The first consists of implanting autologous uterine segments on to the peritoneum, whereas in the second, human endometriotic implants are sutured to the peritoneum of immunocompromised mice [8]–[13]. Both models produce comparable phenotypes, which are then morphometrically evaluated. In a novel, recently described animal model, uterine endometrial fragments from a donor mouse are directly introduced via injection into the peritoneal cavity of an immunocompetent syngeneic recipient. The novel study design additionally enables the evaluation of molecular mechanisms that are critical for disease initiation [14], [15]. We describe our results here in a unique animal disease-model that significantly expands the capability of conducting both mechanistic and translational research in the field.

In this study we identify and describe for the first time a role for the Sp/KLF family transcription factor KLF11 in endometriosis. The ubiquitously expressed Sp/KLF transcription factors regulate most expressed genes by binding to GC-rich promoter domains via their highly conserved Zinc-finger DNA-binding domain [16], [17]. Functionally, KLF9 is the most well characterized Sp/KLF family transcription factor in human uterine endometrium, where it is a progesterone receptor cofactor [18], [19]. KLF9 has a role in endometrial receptivity as well as in cancer [20], [21]. Recently KLF11, a paralog of KLF9, was found to be diminished in uterine fibroids, where it was shown to have a progesterone-dependent, growth suppressive role [22], [23]. KLF11 is also a well-established human disease-associated gene that is etiologically implicated in severe variants of human diabetes, in several cancers as well as in the regulation of diverse endocrine/metabolic pathways [24]–[28]. As uterine endometrium is highly responsive to and regulated by sex steroids, we hypothesized that KLF11 had a role in endometrial biology and disease.

We evaluated KLF11 expression in endometrial tissue obtained both from the uterus as well as from ectopic endometriotic lesions. As we found KLF11 to be diminished in endometriosis tissue, we further investigated its role in disease pathogenesis *in vivo* in *Klf11-/-* mice. To confirm that the observed endometriotic phenotype in these knockout animals was specifically due to loss of Klf11-mediated transcriptional regulation, we also induced endometriosis in *Klf9-/-* animals. This approach enabled comparative investigation of the effect of loss of each specific paralogous protein on endometriotic implants. Therefore, using a robust, combinatorial *in vitro* and *in vivo* approach, we not only identify a novel pathogenic role for *KLF11* in endometriosis, but also characterize the phenotypic consequences that arise out of dysregulated transcriptional regulation from aberrant expression of this gene.

## Materials and Methods

### Ethics Statement

All animal experiments were performed per the recommendations outlined in the Guide for Care and Use of Laboratory Animals from the National Institutes of Health as required by Mayo Clinic. These guidelines were incorporated into the current study protocol (#A4711), which was submitted, reviewed and approved by the Institutional Animal Care and Use Committee (IACUC), at Mayo Clinic, Rochester, MN. All animal experiments were performed after approval of this study (A4711) by the Mayo Clinic IACUC. Animal suffering was minimized with the use of Ketamine and Xylazine anesthesia for surgery. Archived human endometrial and endometriosis samples for tissue microarray were selected per Mayo Clinic Institutional Review Board (IRB) protocol: 11-003074.

### Tissues and Cell lines

Pooled organ-specific human tissue RNA was purchased (Agilent Biotechnologies, USA [pancreas, uterus] or Ambion, Life Technologies, USA [other tissues]) and used for PCR after cDNA synthesis. Ishikawa is an established endometrial adenocarcinoma cell line that is well characterized and commercially available through Sigma-Aldrich, MO [29], [30]. Primary immortalized endometrial stromal cells (T-HESC) are also a well-characterized, established cell line, available commercially from ATCC (#CRL-4003), Manassas, VA [31]. Ishikawa and T-HESC cells were grown in DMEM and DMEM/F-12 respectively, each supplemented with 10% FBS.

### Tissue Microarray Design

Patients with a histologic diagnosis of endometriosis at hysterectomy were selected from the institutional archives of Mayo Clinic's Department of Laboratory Medicine and Pathology for creation of tissue microarrays (TMA) per Mayo Clinic Institutional Review Board (IRB) protocol: 11-003074. Following diagnostic confirmation and adequacy of tissue by review of hematoxylin and eosin (H&E) slides, corresponding formalin-fixed paraffin-embedded tissue blocks were selected for TMA construction. A matched pair of 0.6 mm cores was obtained from each case, one of endometriosis and one of the same patient's endometrium (N: 28).

### PCR and qPCR

Total RNA from one 10-cm dish of 80% confluent Ishikawa or T-HESC cells was extracted according to the manufacturer's instructions using an RNeasy kit (Qiagen) to evaluate cell-type specific *KLF11* expression. 1 µg of organ-specific mRNA was used for comparative analysis. Oligo-dT primer was used for cDNA synthesis in a SuperScript™III first-strand synthesis system for RT-PCR (Invitrogen), per manufacturer's protocol. RT-PCR was performed using Platinum-*Taq* DNA polymerase (Invitrogen) per manufacturer's protocol using a *KLF11* primer with amplification of *GAPDH* as control. Primer sequences are provided in Table 1. Each experiment was done in triplicate. For real time PCR, commercially available gene-specific primers (RT^2^ qPCR Primer Array, Qiagen, USA) were used. The reactions were performed using the IQ-SYBR Green Supermix (Bio-Rad) per the manufacturer's protocol in a CFX-96 Thermocycler (Bio-Rad). All qPCR measurements were carried out within the linear amplification range [32], [33].

**Table 1:** Primer Sequences used for amplification of promoter sequences (ChIP) or expression studies (PCR).

Name	Type	Forward	Reverse
*COL1A1*	Promoter	5′-TCTGGAGCACAGCAGAAGAA-3′	5′-AGCTAAGGGAGGGGCATAAA-3′
*COL1A2*	Promoter	5′-GCCCTTTCCCGAAGTCATA-3′	5′-GATCAAAGCAGGAGCTGTCC-3′
*COL3A1*	Promoter	5′-AAATGGGCATCAAGCAGTTT-3′	5′-CCATCCCCTCAGCAGTAAAA-3′
*MMP3*	Promoter	5′-GACTATAGCTATGTATGTAC-3′	5′-CACTGGCTTTACTTAGCTC-3′
*MMP10*	Promoter	5′-GCTATTGTAAACAAGGACTC-3′	5′-GCCCTTACCTTCTTTGTC-3′
*TGFβR1*	Promoter	5′-GGAGCAGGAGGAAATAGGAG-3′	5′-TCTGTGTGAGTCTCTTTCGG-3′
*KLF11*	Gene	5′-AGACTTTTCCCGAAGGAGGA-3′	5′-TCCGAACGAGCAAACTTTTT-3′
*GAPDH*	Gene	5′-ACCACAGTCCATGCCATCAC-3′	5′-TCCACCACCCTGTTGCTTGTA-3′

### Western Blot Analysis

Total cell lysate was obtained from one 10-cm cell culture dish of Ishikawa and T-HESC cells for western blot analysis. Standard western blot techniques were used to determine protein expression in these uterine cell lines [33]. The antibodies used were primary: Anti-KLF11 (1∶500; Abnova, clone 8F4) and Anti-α-Tubulin (1∶1000; Sigma-Aldrich, MO, USA). The ECL-Plus Western Blotting Detection kit (GE Healthcare, Buckinghamshire, UK) was used for detection. KLF11 was detected as a 65 KDa band and α-Tubulin at 50 KDa.

### Immunohistochemistry

Tissue sections were de-paraffinized and rehydrated in xylene and a graded series of ethanol solutions respectively. Epitope retrieval was performed by heating the slides in a steamer for 20 min in 10 mM citrate buffer (pH 6.0). Endogenous peroxidase was quenched using hydrogen peroxide/methanol followed by Avidin/Biotin blocking (Vector Labs), initial incubation with blocking solution (CAS Block: Invitrogen) followed by incubation with Anti-KLF11 (Abnova, clone 8F4: 1∶100 dilution) overnight at 4 °C. Sections were incubated with secondary antibody alone as a negative control. The sections were incubated with a 1∶500 dilution of secondary biotinylated horse anti-mouse antibody (Vector Labs) for 30 min at ambient temperature followed by Streptavidin (Invitrogen). Nova Red (Vector Labs) and Mayer′s hematoxylin were used as counterstains. Histochemical staining for collagen deposition using Masson′s trichrome in mouse endometriosis implants was performed by the Mayo Pathology Department Core Laboratory. Examination of the stained sections was conducted using a Nikon Labophot-2 microscope and Image-Pro Plus 5.0.1 acquisition software (Media Cybernetics, Bethesda, MD, USA). Two independent pathologists evaluated staining. The glandular epithelium and stroma of each core were evaluated for stain intensity and extent. Qualitatively, staining was scored as none, weak, moderate or strong and assigned scores of 0, 1, 2 and 3 respectively; extent of staining was then evaluated by determining the percentage of cells with different staining intensities. The extent-percentages were then multiplied by respective staining intensity-scores to obtain a composite H-Score, as previously described [34].

### *Klf9 and 11-/-* mice

The KLF11 knock- out model was generated at the University of Washington, Seattle, following standard homologous recombination techniques (21). This animal, at the time of generation had a mixed background and this was subsequently crossed back into a pure C57BL/6 background for more than 20 generations after transfer to the Mayo Animal Facilities, wherein the inbred strain used in this study was eventually generated. In all experiments, *Klf11-/-* animals were compared with wildtype *Klf11+/+* (wt) littermates. *Klf9-/-* animals were deposited in the Jackson Laboratory by Rosalia Simmen Ph.D. (University of Arkansas Medical Center, Little Rock, USA) [21]. The Urrutia Laboratory obtained a breeding pair of these animals from Jackson Labs and their progeny were used for these experiments.

### Mouse Endometriosis Model

Endometriosis was surgically induced in 8 week old *Klf11-/-, Klf9-/-* and control wild type animals (N = 7 animals in each group) using a well-characterized surgical approach that comprised of resection of one complete uterine horn followed by autologous transplantation of two 5 mm everted segments by suture to the parietal peritoneum [8], [10], [11], [35]–[37]. Eversion of the uterine horns ensured that the endometrial aspect of each resected uterine segment faced the peritoneal cavity. The animals were evaluated at necropsy 3 weeks after initial implantation for lesion assessment. All animal experiments were performed as outlined in the study protocol (#A4711), which was submitted, reviewed and approved by the Institutional Animal Care and Use Committee (IACUC), at Mayo Clinic, Rochester, MN. Animal suffering was minimized with the use of Ketamine and Xylazine anesthesia for surgery.

### Luciferase Assay

The pGL3-basic empty vector was purchased (Promega, USA) and pGL3 basic promoter-reporter constructs containing elements from *Collagen1A1, 1A2, 3A1, MMP3, MMP-10 and TGFβR1* were generated in the Urrutia laboratory. The T-HESC cell line (∼80% confluent) was co-transfected with either 2.5 µg of pcDNA3/His empty vector (Invitrogen, USA) or a pcDNA3/His-KLF11 construct and 3 µg of each pGL3 promoter-reporter construct. Forty-eight hours after transfection, cells were lysed and reporter activity was read using the Luciferase assay system (Promega, USA) and a 20/20 luminometer (Turner Designs, USA), according to manufacturer′s protocol. Data in relative light units were normalized to lysate protein concentrations and shown as the mean ± standard error of the means. Experiments were performed in triplicate, three independent times.

### Chromatin Immunoprecipitation

ChIP assay was performed as previously described [33]. Briefly, uterine stromal cells (T-HESC) were grown to confluence and 10 million cells lysed for further analysis. DNA shearing was performed to produce fragments 200 – 600 base pairs in size. IP was performed using Anti-KLF11 or control rabbit IgG. PCR products representing the *COL1A1, COL1A2, COL3A1, MMP3 and TGFβR1* promoters containing putative KLF11-binding GC elements were examined on a 2% agarose gel. All primer sequences are provided in Table 1.

### Statistical Analysis

All results are expressed as means with standard error of means (SEM). Each experiment was repeated in triplicate at least three times. Densitometric comparison of PCR product was done using Image Processing and Analysis in Java (Image J) software available from the NIH. F-test of treatment mean equality and Bonferroni method of multiple comparisons (t tests) or the χ^2^ test were used, as applicable. Statistical analysis was performed using SAS software (SAS Institute, Cary, NC). For consistency, all p values are reported as <0.05, where significant, throughout. All statistical tests were two-sided.

## Results

### KLF11 was selectively expressed at high levels in urogenital tissues and in endometrial cells

**Figure 1:**
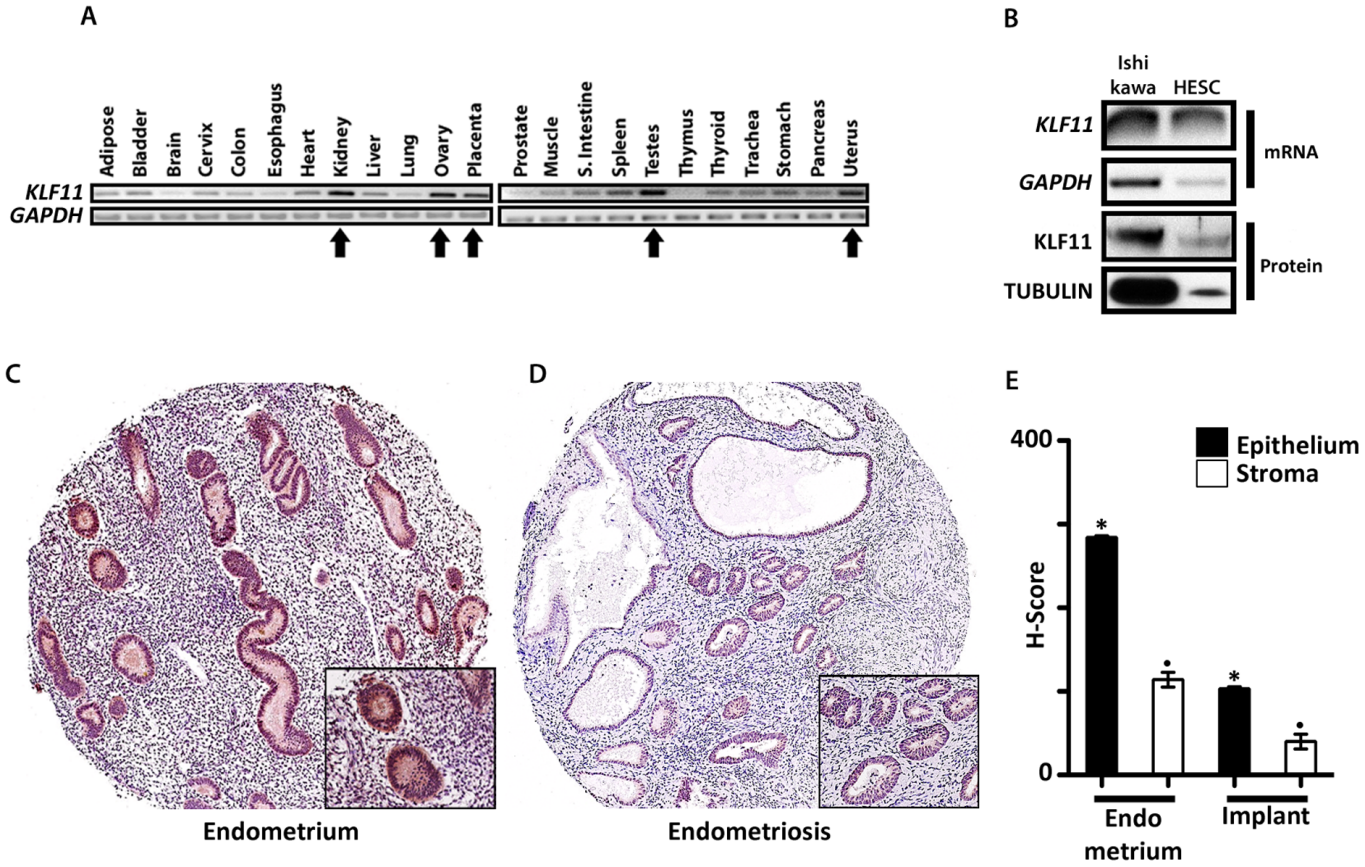
KLF11 expression in human urogenital tissues, uterine eutopic endometrium and endometriosis. (**A**) Pooled Human Organ-specific RNA (1 µg/reaction) was analyzed for *KLF11* mRNA expression by PCR. mRNA levels of the housekeeping gene *GAPDH* were simultaneously assessed as loading control. Relative organ-specific *KLF11* mRNA expression levels were determined by densitometric comparison to corresponding *GAPDH* levels. KLF11 mRNA expression levels were increased in urogenital tissues such as the kidney, ovary, placenta, testes and uterus (Figure 1A, arrows) compared to other non-urogenital tissues. (**B**) Expression of KLF11 mRNA and protein were also assessed in two cell lines Ishikawa, a well-differentiated endometrial adenocarcinoma cell line and Human Endometrial Stromal Cells (T-HESC), which are frequently used as model endometrial epithelial and stromal cell lines respectively. *KLF11*/KLF11 were expressed in both cell-lines; *GAPDH/*α-TUBULIN were used as a reference controls. To qualitatively demonstrate KLF11 expression in each of these endometrial cell lines, lysate for RNA or protein extraction was obtained from one 10-cm cell culture dish of confluent cells. Due to much lower abundance of stromal compared to epithelial cells there appears to be differential *KLF11*/KLF11 expression between the cell-types. However, this difference was also reflected in expression of the loading controls *GAPDH*/α-TUBULIN between the two cell lines. (**C, D**) KLF11 expression was also evaluated by immunohistochemistry in eutopic endometrium as well as in ectopic endometrial implants (magnification: 200×: panel; 400×: inset). KLF11 was expressed in the nuclei and cytoplasm of epithelial as well as stromal cells in both eutopic endometrium as well as in endometriotic implants. KLF11 expression was diminished in endometriotic implants compared to eutopic endometrium. Representative samples shown. (**E**) KLF11 expression levels were compared and scored in paired eutopic endometrium and ectopic endometriotic implants obtained from the same patient (N = 28 paired samples). The expression was significantly reduced in the implants (epithelium: 103±2.6; stroma: 40.4±3.9) compared to that in eutopic uterine endometrium (epithelium: 278±2.5; stroma: 114±8.7). * and. = p<0.05 and represent comparisons between epithelial and stromal cells in eutopic and ectopic endometrium respectively.

The 24 Sp/KLF transcription factors are ubiquitously expressed in the body; one mechanism for specificity of transcriptional regulation is via tissue-selective expression of distinct paralogs. To assess whether KLF11 expression was organ-specific, we evaluated KLF11 mRNA expression across an array of human tissues. The relative mRNA expression levels were highest in gonadal tissues such as the ovaries and testes as well as in other urogenital tissues such as the uterus, placenta and kidneys (Figure 1A, arrows). Human *GAPDH* was used as a loading control. KLF11 mRNA and protein were expressed in Ishikawa and Human Endometrial Stromal Cell (T-HESC), which are frequently used as model endometrial epithelial and stromal cell lines respectively (Figure 1B) [29], [31]. Due to much less abundant numbers of stromal compared to epithelial cells per equivalent growth surface area, expectedly less RNA and protein lysate were obtained from the former cell-type. This difference is reflected in the differential expression of the control housekeeping gene *GAPDH/*TUBULIN between the two cell lines (Figure 1B).

### KLF11 expression was diminished in ectopic endometriosis implants compared to eutopic uterine endometrium

In order to determine if KLF11 may have a role in endometriosis, we used immunohistochemistry to evaluate its expression in a TMA consisting of paired eutopic uterine endometrium (Figure 1C) and ectopic endometriotic implants (Figure 1D) from 28 patients. H-scores quantifying the intensity and extent of KLF11 expression in these tissues were compared [34]. KLF11 expression levels in both epithelial and stromal cells were significantly diminished in endometriosis lesions compared to eutopic endometrium (representative samples, Figure 1C, D). Consistent with these findings, the immunohistochemistry H-scores for epithelial (278±2.5) and stromal cells (114±8.7) in eutopic uterine endometrium were significantly greater than corresponding scores in ectopic endometriotic implants (epithelium: 103±2.6; stromal: 40.4±3.9; * and. = p<0.05 and represent comparisons between epithelial and stromal cells respectively in eutopic and ectopic endometrium) (Figure 1E).

### *Klf11-/-* mice had large peritoneal lesions with prolific fibrosis resembling advanced human endometriosis

**Figure 2:**
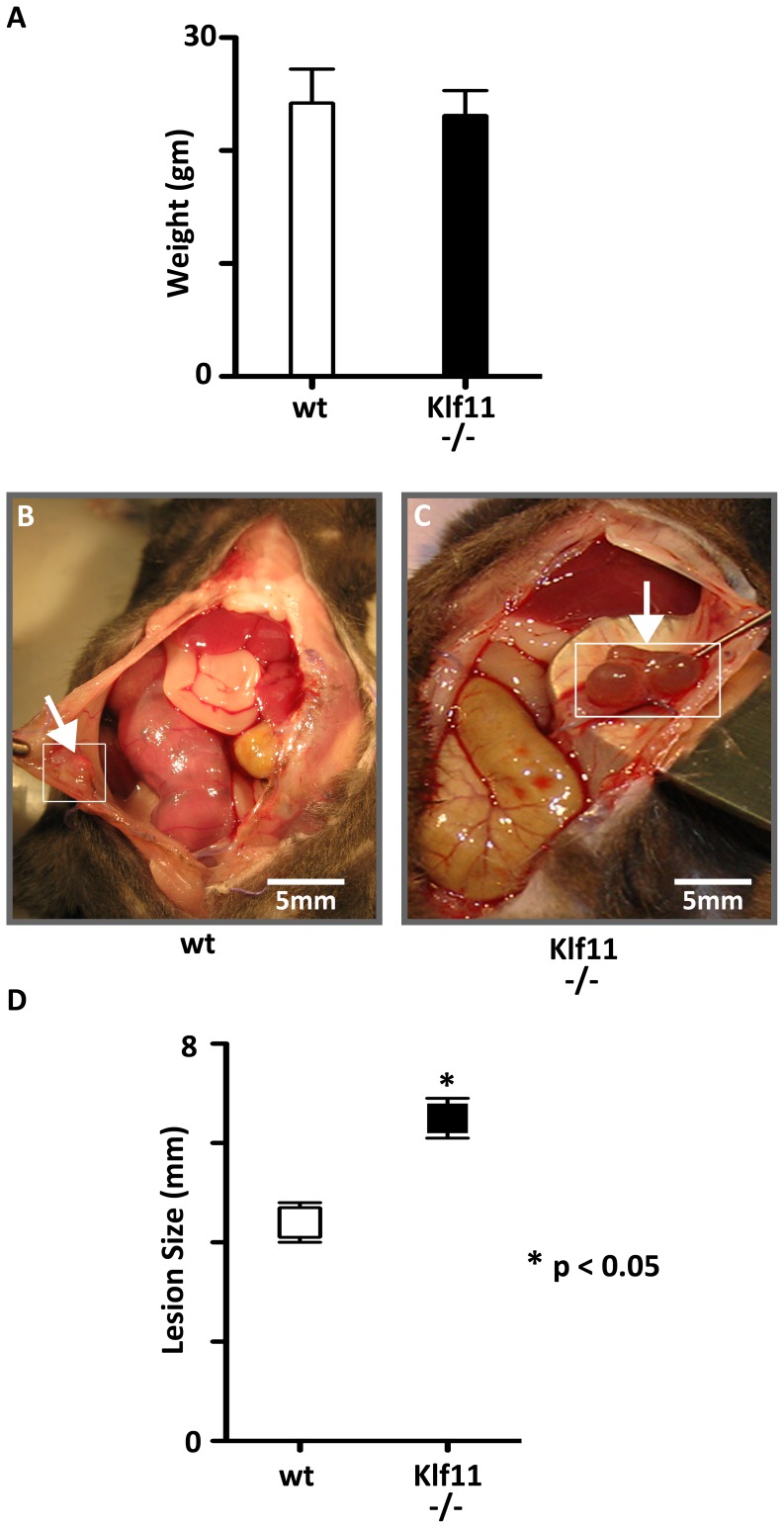
Role of Klf11 on lesion size in endometriosis. (**A**) Endometriosis was surgically induced in 8 week old *Klf11-/-* and wildtype female mice (N = 7/group). All animals were weighed prior to initial surgery and at necropsy 3 weeks later. There was no difference in weight between the groups either prior to implantation surgery or before subsequent necropsy. Comparative weight profile prior to necropsy shown (p>0.05; NS). (**B, C**) At induction, 0.5cm endometrial implants were implanted on to the parietal peritoneum of *Klf11-/-* and wildtype mice. Lesion size was evaluated at necropsy three weeks after initial surgery. The peritoneal lesions (white arrows; box) in *Klf11-/-* mice (C) were larger and more cystic compared to those observed in wildtype controls (B). (**D**) The lesions in *Klf11-/-* animals (6.8±0.044mm) were significantly larger than those observed in wildtype controls (4.5 ± 0.029mm). [*  = p<0.05; 14 lesions per genotype].

**Figure 3:**
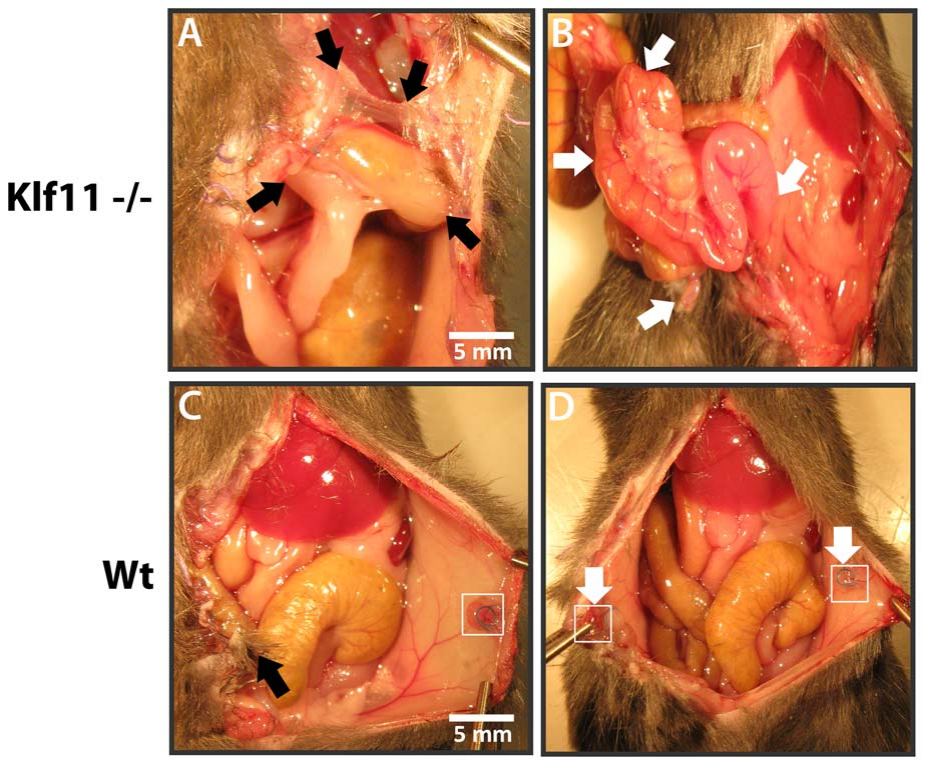
Role of Klf11 in endometriosis-associated fibrosis. Endometriotic lesions in *Klf11-/-* mice were associated with prolific *de novo* scar tissue formation in contrast to wildtype controls. (**A**) Adhesions in *Klf11-/-* animals were thick, opaque, dense and unyielding to mechanical disruption by pressure. The adhesions had a broad base (black arrows) and involved adjacent viscera such as the small and large intestine, stomach and liver, thereby resulting in obliteration of physiological tissue planes. (**B**) Progressive fibrosis further involved the intestinal mesentery in these animals, resulting in straightening of the bowel with apparent shortening of length (white arrows). (**C**) In contrast, in wildtype animals, the lesions remained discrete (white arrows and box in C and D) with minimal adhesions (black arrow). Any adhesions that formed were slender, transparent, non-obliterating and very easily disrupted by pressure. (**D**) Lack of progressive and prolific fibrosis in wildtype animals preserved normal intra-abdominal anatomy with no peritoneal obliteration or mesenteric fibrosis.

**Figure 4:**
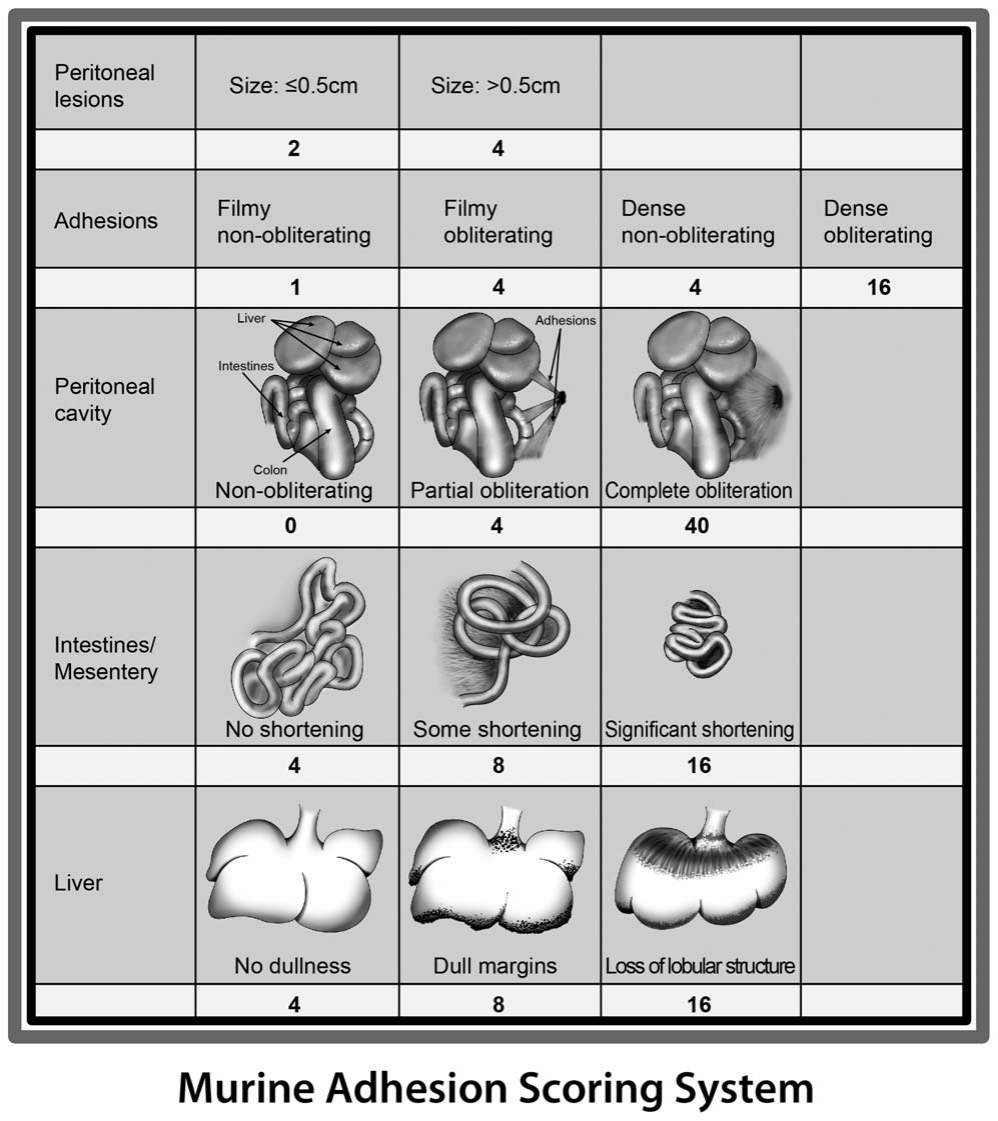
Application of a Novel Fibrosis Adhesion Scoring System to evaluate murine endometriotic lesions. A murine peritoneal sclerosis scoring system was modified and adapted to the revised ASRM endometriosis staging system. Accordingly, anatomic landmarks in the region of the lesions were incorporated and weighted scores were assigned in accordance with the established human disease scoring system.

**Figure 5:**
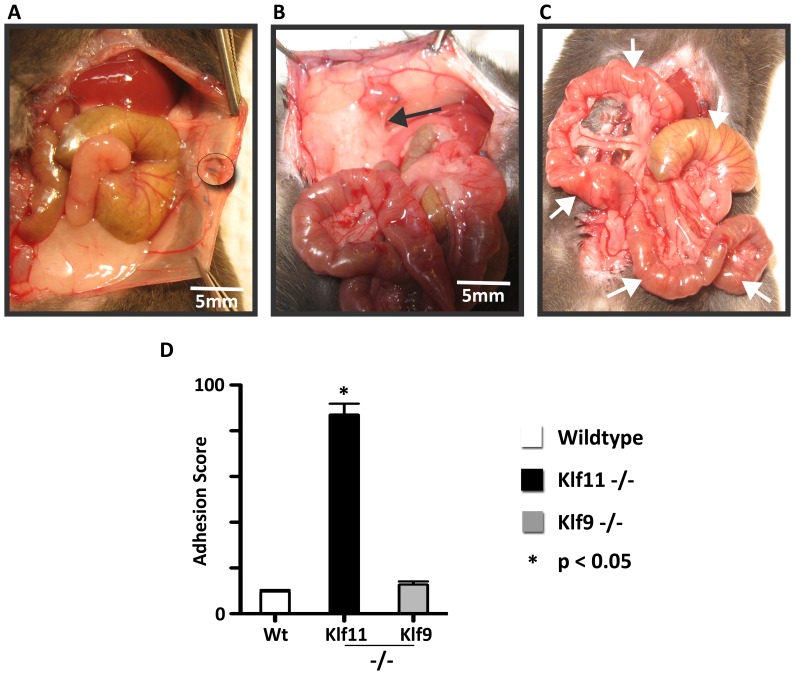
Comparison of the role of Klf9 and Klf11 in an animal endometriosis model. (**A**) Endometriotic Lesions (circled) in *Klf9-/-* animals were either unchanged or had regressed in size from the time of initial peritoneal implantation, when evaluated at necropsy 3 weeks later. (**B**) Endometriotic lesions in these animals also did not elicit a progressive fibrotic response as seen in *Klf11-/-* animals. Any adhesions in *Klf9-/-* animals (black arrow) were flimsy, transparent and peri-lesional in extent. (**C**) Tissue planes were unaltered with preservation of intra-abdominal anatomy. Consequently, intestinal length was not foreshortened due to lack of mesenteric fibrosis (unraveled intestinal loops denoted by white arrows). (**D**): A composite adhesion score for each mouse was determined and compared between *Klf11-/-*, *Klf9-/-* and wildtype genotypes, based on the Murine Adhesion Scoring System. The adhesion score for *Klf11-/-* mice (81.7±4.8) was significantly different from that calculated in either *Klf9-/-* (12.3±1.8) or wildtype animals (9.17±0.8); (* = p<0.05, 14 lesions/genotype). The scores objectively reflected observed anatomical findings in these animals.

As KLF11 expression was diminished in endometriotic implants compared to uterine endometrium, to further characterize the specific role of KLF11 in endometriosis *in vivo*, we investigated its effect on disease pathogenesis in a *Klf11-/-* knockout mouse model. Endometriosis was surgically induced in 8-week old female mice. The resultant phenotypes in *Klf11-/-* animals were compared with those in simultaneously treated wild-type animals (N = 7/group) (Figure 2). There was no difference in weight in either *Klf11-/-* or wildtype animals either prior to implantation surgery or before subsequent necropsy (Figure 2A). There was also no evidence of ascites in either group of animals. The peritoneal lesions in *Klf11-/-* mice were significantly larger (6.8±0.044 mm) compared to those observed in wildtype controls (4.5 ± 0.029 mm); p<0.05, (Figure 2 B-D). Further, the lesions were cystic in *Klf11-/-* mice in contrast to small, involuting lesions in wildtype animals (Figure 2B, C: arrows). The lesions in *Klf11-/-* mice were significantly associated with extensive, dense adhesions involving the small intestine, colon, stomach and liver (Figure 3A, B). In most cases, the adhesions completely encased the endometriotic implant, thereby necessitating extensive dissection in order to identify the implant. The adhesions were broad-based, resistant to mechanical disruption and resulted in widespread peritoneal fibrosis rather than limited peri-lesional scarring (Figure 3A, arrows denote bridging scar between lesions). Widespread, progressive fibrosis also involved the mesentery resulting in straightening of the intestinal loops, which was evident phenotypically as apparent shortening in length (Figure 3B: arrows). In contrast to *Klf11-/-*, in wildtype animals, the lesions were discrete, unchanged or regressed in size and occasionally associated with flimsy, transparent adhesions that could easily be disrupted with pressure (Figure 3C, D: arrows). As the endometriotic phenotype resembled advanced human disease, we modified a murine peritoneal-fibrosis scoring system to adapt to the revised American Society of Reproductive Medicine (ASRM) endometriosis staging criteria (Figure 4) [38], [39]. We used this murine endometriosis scoring system to quantify the disease-phenotype, as is clinically done in human endometriosis. Using this system, the mean adhesion score for *Klf11-/-* animals was significantly greater (81.7±4.8) compared to that in wildtype (9.17±0.8) animals; p<0.05 (Figure 5D).

### *Klf9-/-* animals had minimal endometriotic implant-associated growth and fibrosis

Klf9 is the most extensively characterized Sp/Klf paralog in the endometrium. *Klf9-/-* mice have defects in endometrial receptivity resulting in diminished litter-size, which is however partly phenotypically compensated for by overexpression of a related paralog [21]. In order to evaluate the specificity of *Klf11* in the pathogenesis of endometriosis, we also surgically induced endometriosis in *Klf9-/-* mice (Figure 5). Similar to wildtype, and in contrast to *Klf11-/-* animals, lesions in *Klf9-/-* animals were either unchanged or regressed in size (Figure 5A). The extent of fibrosis was also minimal with occasional, flimsy adhesions that could be easily dissected off the lesion using minimal mechanical pressure (Figure 5B). No mesenteric scarring causing apparent intestinal shortening was evident (Figure 5C). *Klf9-/-* animals therefore did not develop the widespread fibrotic response in contrast to the phenotype observed in *Klf11-/-* animals. Their lesion size (4.3±0.2mm) was similar to wildtype animals. The peritoneal fibrosis score (12.3 ± 1.8) in these animals calculated using the murine adhesion scoring system (Figure 4) was also similar to that in wildtype animals, although significantly different from that obtained in *Klf11-/-* animals (Figure 5D).

### KLF11 regulated fibrosis-associated gene signaling networks in endometriosis

**Figure 6:**
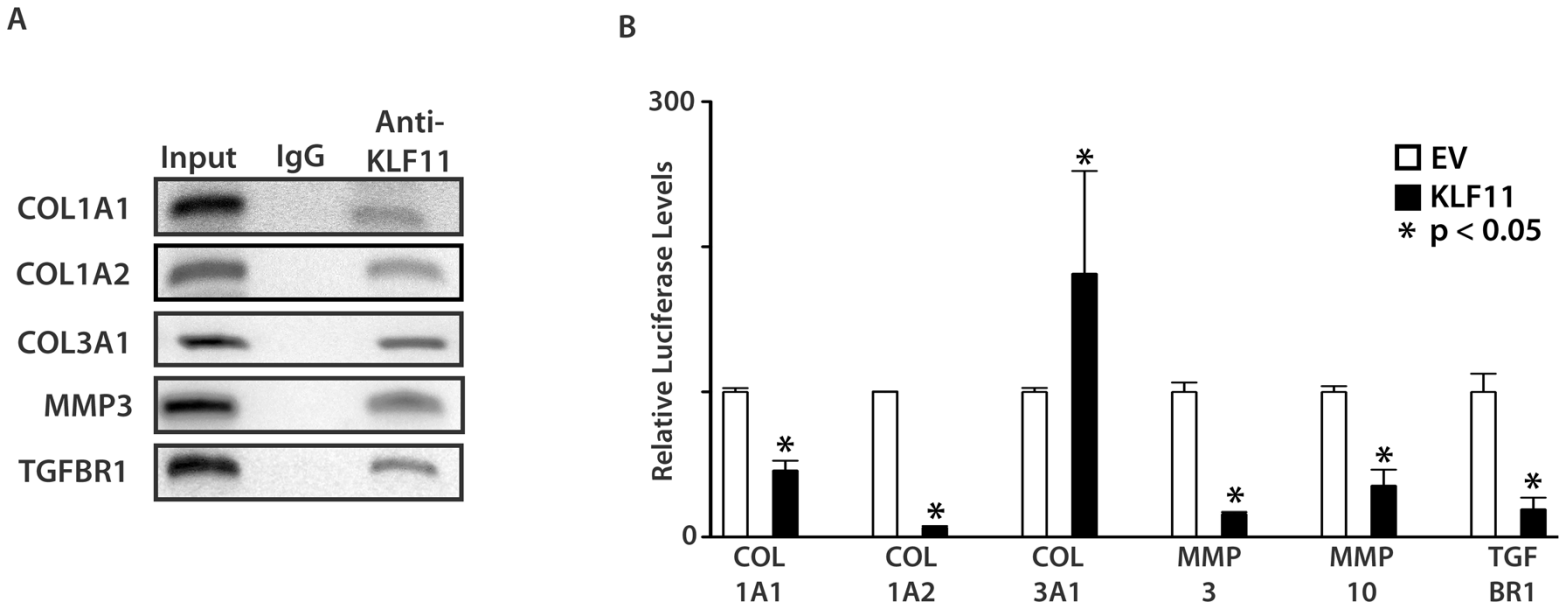
Evaluation of the Role of KLF11 in the regulation of fibrogenic signaling. (**A**) KLF11 binding to the promoters of known fibrosis associated genes was evaluated by Chromatin immunoprecipitation *in vivo* in endometrial stromal cells. Representative targets from diverse families of fibrogenic genes are shown (Collagens, MMP, TGF-β signaling pathway). KLF11 but not a control species and isotype-specific IgG bound candidate promoter GC-elements in these putative target genes. (**B**) Functional competence of the KLF11-binding promoter element was tested in promoter-luciferase reporter assays. T-HESC endometrial cells were transfected with 2.5 µg of either pcDNA3/His-*KLF11* or corresponding pcDNA3/His-*EV* and 3 µg of pGL3/promoter-reporter construct for 48 hours. Normalized luciferase expression levels obtained with KLF11 compared to EV are shown. Compared to corresponding empty vector, KLF11 significantly repressed COL1A1, 1A2, MMP3, 10 and TGFβR1- promoter luciferase levels, whereas it activated expression from the COL3A1-reporter (* = p<0.05).

**Figure 7:**
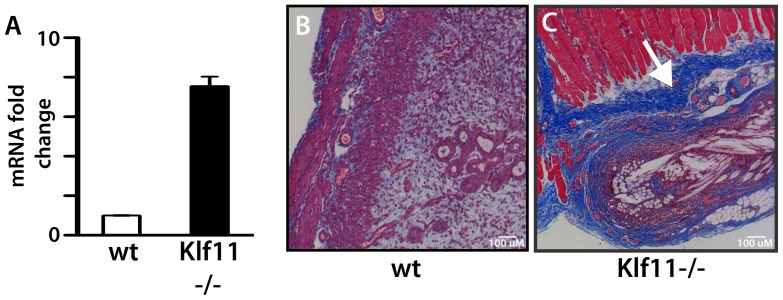
Role of Klf11 in the regulation of Col1a1 expression. (**A**) Collagen 1A1 mRNA expression levels were determined from one of two endometriotic implants in each animal (*Klf11-/-* and wildtype). *Col1a1* mRNA expression levels were increased 7.55±0.48 fold (p<0.05) in implants from *Klf11-/-* animals compared to wildtype. (**B, C**) Histochemical evaluation using Masson′s trichrome staining showed that collagen deposition (blue stain, white arrow) was increased in the tissue surrounding peritoneal endometriosis implants in *Klf11-/-* animals (C) in contrast to wildtype controls (B) Magnification: 100×.

Loss of Klf11 results in a prominent fibrotic response in association with induction of endometriosis. Widespread fibrosis is frequently seen in advanced human endometriosis. We therefore focused on further mechanical characterization of this specific phenotype, and consequently investigated the regulation of fibrotic signaling by Klf11 *in vitro* in endometrial stromal cells. We initially identified putative GC-rich KLF binding elements bioinformatically in the promoters of an array of genes involved in fibrosis, such as various collagen proteins, matrix metalloproteinases (MMP), their tissue inhibitors and mediators of the pro-fibrotic TGF-β signaling pathway. Using Chromatin immunoprecipitation, we found that KLF11 bound these elements in endometrial cells. Representative examples: *COL1A1*, *COL1A2*, *COL3A1*, *MMP-3* and *TGFβR1* are shown (Figure 6A). We next evaluated transcriptional regulation of these genes by KLF11 in promoter-luciferase reporter assays (Figure 6B). Whereas, KLF11 repressed transcription from the Collagen 1A1, 1A2, MMP-3, MMP-10 and TGFβR1 promoters, it activated Collagen 3A1 promoter-reporter expression (Figure 6B). Collagen 1 is the predominant Collagen protein in *de novo* scar tissue. *In vitro* repression of Collagen 1 in promoter-reporter assays suggested that absence of Klf11 in knockout animals could likely de-repress and thereby activate Collagen 1 expression. A prominent phenotype of our endometriosis disease model in *Klf11-/-* mice was prolific scarring, possibly due to lack of repression of Collagen 1. We therefore evaluated Collagen 1 expression levels in endometriotic implants obtained from *Klf11-/-* and wildtype animals. The mRNA expression levels for the *Col1A1* isoform was increased (7.5 fold; p<0.05) in implants from *Klf11-/-* mice in comparison to wild-type animals (Figure 7A). The increased mRNA expression was accompanied by increased Collagen 1 deposition in and around endometriotic implants in *Klf11-/-* animals in contrast to wildtype (Figure 7B, C: blue staining).

## Discussion

In this study we investigated the role of KLF11 in endometriosis, a common, debilitating, endocrine hormone driven female urogenital disorder. We found that expression levels of KLF11 were diminished in human endometriotic implants compared to eutopic endometrium. Our studies further showed that selective and specific loss of *Klf11*, in contrast to *Klf9*, was associated with the development of a characteristic phenotype that resembled advanced human endometriosis in a murine disease model. Accordingly, in *Klf11-/-* animals the lesions were significantly larger and cystic compared to those in wildtype controls as well as in *Klf9-/-* mice. Additionally, the lesions in *Klf11*-/- animals were associated with dense adhesions involving surrounding viscera such as the small and large intestine, stomach and liver. KLF11 also repressed key fibrotic-signaling associated genes, thereby corroborating the observed phenotype. The data therefore suggests that KLF11 has a pathogenic role in endometriosis resulting in lesion growth and prolific fibrotic scarring. Widespread adhesions and fibrosis are commonly found in patients with advanced endometriosis and are associated with chronic pain and pelvic morbidity. We therefore focused this study on mechanistic investigation of this disease related phenotype.

We modified and adapted a murine peritoneal adhesion scoring system to the revised American Society of Reproductive Medicine (ASRM) endometriosis staging system in order to quantify the extent of disease as is currently done in clinical practice [39]. Accordingly, weighted lesion-associated scores were assigned to reflect those used in the clinical staging system. The current ASRM staging system is easy to implement, reproducible, amenable to objective evaluation of disease extent as well as for assessing treatment-response. We optimized the use of this widely accepted, established paradigm to objectively evaluate pathogenesis in our disease model (Figure 4). The ASRM staging system has been criticized for having limited prognostic capability, as it is not based on disease etiology [40], [41]. In this study however, we employed the staging system solely for objective determination of disease extent in order to enable comparison amongst different animals, with no further prognostic extrapolation. This approach has enabled us to use the system effectively not only for objectively evaluating the effect of loss of Klf11 or Klf9 on the pathogenesis of endometriosis, but also to investigate the effect of planned environmental and therapeutic interventions designed to arrest this process.

The varied pathology and clinical presentations of endometriosis reflect the underlying diversity in disease etiopathogenesis. Consequently, an array of disrupted molecular mechanisms such as genetic and epigenetic signaling networks as well as inflammatory and immunological mechanisms, amongst others may be implicated. Whereas cell-based *in vitro* experiments provide a framework for testing molecular mechanisms, eventual confirmation of their role in disease causality *in vivo* can only be provided by a suitable animal model. For a disease as diverse as endometriosis, it is unlikely that a single animal model would be sufficient to represent the entire diversity in the etiology, pathogenesis and pathology. Each model is expected to have design-related strengths and limitations. For example, a recently described disease model consists of introduction of endometrial tissue via injection into the peritoneal cavity of immunocompetent mice [14], [15]. This model physiologically replicates retrograde menstruation, the most widely accepted theory of endometriosis development. Consequently, the model is most suited for investigating the establishment of endometriotic implants. In contrast, the model used in the current study evaluates later events that sequentially follow implant-establishment and are prominent in chronic, symptomatic disease. Each stage in the pathogenesis of endometriosis most likely has uniquely disrupted signaling mechanisms that are best evaluated by appropriate choice of model design. The strength of both of these models is the adaptability of use in specific genetic backgrounds such as Klf11-/-, and receptor knockouts for Leptin, ERα and β [14], [15]. Such a strategy permits linkage between the underlying disrupted genetic pathway and the resultant abnormal disease phenotype. For example, in the current study the Klf11-/- genetic background allowed us to define a novel role for this human disease-related gene in endometriosis in contrast to its paralog Klf9. In future studies, this model is expected to be critical for studying the impact of disrupting epigenetic and cell signaling pathways that are intrinsically linked to transcriptional regulation by KLF11.

KLF11 is a member of the widely expressed Sp/KLF family of zinc-finger transcription factors. These proteins bind to GC-rich regions in the promoters of their target genes. GC-rich regions are enriched in transcriptionally active regions of the genome, and are therefore collectively the most ubiquitous response elements [42]–[44]. The combination of a high prevalence of binding sites capable of binding at least one of 24 Sp/KLF-paralogs enables these transcription factors to regulate almost all expressed genes. Therefore, one mechanism of overcoming functional redundancy amongst the paralogs is via tissue-selective expression of specific family members. In this study, we found that KLF11 specifically was expressed at high levels in urogenital tissues.

KLF9, a paralog of KLF11 has been extensively characterized in endometrial cells. KLF9 is a cofactor of both progesterone receptor isoforms and co-operatively regulates endometrial proteins such as uteroferrin [19]. Loss of Klf9 is associated with diminished fertility, embryo implantation defects and endometrial cancer [20], [21]. To determine specificity of the endometriotic phenotype in *Klf11-/-* animals, we comparatively investigated the phenotype in *Klf9-/-* animals and found discordance. It is therefore unlikely that Klf9, an endometrium-specific paralog compensated for loss of Klf11 or vice versa in our disease model system.

On binding to its cognate target promoter element, KLF11 recruits nuclear cofactor complexes. These cofactor complexes are either coactivators such as CBP/p300 or corepressors such as Sin3a and HP1 [26], [45], [46]. Each cofactor complex contains a specific histone-modifying enzyme, which is a deacetylase or an acetyl or methyl transferase respectively. On recruitment by KLF11 to the vicinity of specific target gene promoters, these enzymes effect their respective post-translational modifications resulting in localized histone deacetylation, acetylation or methylation respectively. The resultant histone modifications in turn alter the chromatin configuration of the target gene promoter, which affects gene expression. For example, histone deacetylases and methyltransferases mostly cause chromatin condensation resulting in displacement of RNA polymerase II and gene silencing. In contrast, histone acetyl transferases affect chromatin expansion resulting in gene activation. It is therefore likely that whereas KLF11 silenced Collagen 1 expression via recruitment of a nuclear corepressor complex, it activated COL3A1 by a coactivator mechanism. Collagen 1 is the most abundant protein in humans and also the predominant Collagen protein in *de novo* scar tissue [47]. Each Collagen 1 molecule is a triple-helix of two Collagen1A1 and one Collagen1A2 fibrils [48]. We found that KLF11 repressed transcription of both Collagen 1 isoforms (Figure 6B, 7A). In this study, we therefore not only demonstrate, the transcriptional regulation of Collagen 1 by KLF11 at the molecular level, but more significantly show the relevance of this signaling mechanism to disease phenotype as is evident from the prolific fibrosis in *Klf11-/-* animals. Pharmacologically targeting this signaling mechanism could therefore provide effective therapy.

Histone-modifying posttranslational mechanisms alter expression levels in the absence of any alteration in gene sequence (e.g. mutation) within a broad epigenetic framework of regulation. In contrast to gene mutations that irrevocably alter gene expression, epigenetic mechanisms are potentially reversible and have been increasingly targeted pharmacologically [49]–[51]. Identification of dysregulation in these molecular mechanisms could therefore generate novel therapeutic targets and thus expand the capability and scope of treatment options for endometriosis. By extension, pharmacologically targeting a dysregulated fibrotic signaling process would be useful in treating other fibrotic diseases as well.

KLF11 is itself also a target for post-translational modification via phosphorylation. For example, KLF11 is phosphorylated at Thr56 by AKT; Phosphorylation at KLF11Thr56 alters its regulation of phospholipase A2, the rate-limiting enzyme in prostaglandin biosynthesis [45]. AKT is an effector of epidermal growth factor (EGF) signaling; KLF11 is therefore also a target of the EGF pathway. The EGF signaling pathway has a key role in cell-survival, and is known to be operative in endometriotic implants [52]–[55]. Finally KLF11 itself, is a TGF-β regulated transcription factor [56]. The TGF-β pathway is pro-fibrogenic and activated in endometriosis [57]–[59]. Therefore, pharmacologically targeting either histone-modifying enzymes, kinases, EGF and/or TGF-β signaling pathways could all potentially alter transcriptional regulation of Collagen 1 by KLF11. Each of these mechanisms therefore constitute additional novel therapeutic targets that could specifically ameliorate pathological scarring.

In summary, we have identified here a novel role of the human disease gene KLF11 in endometriosis. Using an animal model, we have characterized the phenotypic sequelae from dysregulated Klf11 signaling. Our findings suggest that KLF11 not only prevents growth of endometriotic lesions, but via repression of Collagen1, significantly also inhibits pathological scarring, a common finding in patients with advanced disease. Increased treatment efficacy can be achieved by targeting specific dysregulated biological signaling pathways, in contrast to the current empiric approach. Novel therapies that evolve from such translational biomedical research are expected to improve outcomes in a patient-focused, individualized manner.
